# Culture-negative chronic hematogenous osteomyelitis in a two months old girl: a case report

**DOI:** 10.1186/s12891-021-04547-4

**Published:** 2021-08-11

**Authors:** Cheng-he Qin, Rui Tao, Ji-wei Luo, Liang Hong, Lei Xu, Jia Fang, Chun-hao Zhou

**Affiliations:** 1 Department of Orthopaedics and Traumatology, Southern Medical University Nanfang Hospital, No. 1838, Guangzhou Ave. North, Guangzhou, Guangdong 510515 People’s Republic of China; 2 Department of Orthopaedics, Southern Medical University Zengcheng branch of Nanfang Hospital, No. 28, Chuangxin avenue, Yongning Street, Zengcheng District, Guangzhou, 511340 People’s Republic of China; 3 Department of Orthopaedics and Traumatology, Guangdong Second Provincial General Hospital, the Second Clinical Medical School of Southern Medical University, Guangzhou, 510317 People’s Republic of China

**Keywords:** Culture-negative, Chronic hematogenous osteomyelitis, Children, Infection, Pediatric, Case report

## Abstract

**Background:**

Previous articles have focused on the diagnosis and treatment of acute hematogenous osteomyelitis. Here, we present a case of chronic hematogenous osteomyelitis in a 2-month-old girl. The diagnostic procedure was unusual and difficult due to negative culture results.

**Case presentation:**

A girl aged 2 months and 23 days had fever and swelling in her right lower leg for 7 days. On the basis of her medical history, physical, and histological examination results; and radiologic and magnetic resonance imaging findings, a diagnosis of chronic osteomyelitis was made. The patient underwent surgical treatment and was discharged successfully. The patient showed good recovery and no sequelae at the 12-month follow-up.

**Conclusion:**

Hematogenous osteomyelitis in babyhood is different from that at any other age. Hematogenous osteomyelitis-related bone destruction in babyhood is more serious and occurs faster. The transition from acute hematogenous osteomyelitis to chronic hematogenous osteomyelitis takes only 7 days. To the best of our knowledge, this chronic hematogenous osteomyelitis patient is the youngest ever reported.

## Background

Acute hematogenous osteomyelitis (AHO) is an infection of bone tissue by pathogenic microorganisms and represents the most widespread musculoskeletal infection in childhood [1]. Trauma, neoplasm, inflammatory arthropathy, and synovitis may all present with a clinical picture similar to osteomyelitis.*S. aureus* is the most common pathogen of septic knee arthritis and acute hematogenous osteomyelitis [2, 3]. In 20% or more of osteomyelitis cases, no organism is identified, making diagnosis challenging [4]. A majority of pediatric AHO cases can be cured by prompt, appropriate therapy. Ineffective or delayed treatment may result in poor outcomes and progression to chronic osteomyelitis [5]. Acute osteomyelitis is characterized by rapid-onset pain and systemic signs within 2 weeks of the onset of infection and usually no radiographic findings [6, 7]. Subacute osteomyelitis is characterized by insidious-onset pain and absence of systemic signs 2 weeks after the onset of infection [8]. In general, chronic osteomyelitis is characterized by the possible of involucrum formation and sequestra, [6] months after the onset of infection [9]. However, clinical symptoms for more than 10 days are related to the progression of bone necrosis and chronic osteomyelitis [10]. Previous studies have rarely reported culture-negative chronic hematogenous osteomyelitis in children. Some articles reported surgical methods for chronic osteomyelitis in children [11, 12]. However, the ages of the chronic osteomyelitis patients were not mentioned. We report a case of culture-negative chronic hematogenous osteomyelitis that had developed in the distal tibial metaphysis in a 2-month-old girl. To the best of our knowledge, this is the first published case report of culture-negative chronic osteomyelitis in a patient this young.

## Case presentation

**Fig. 1:**
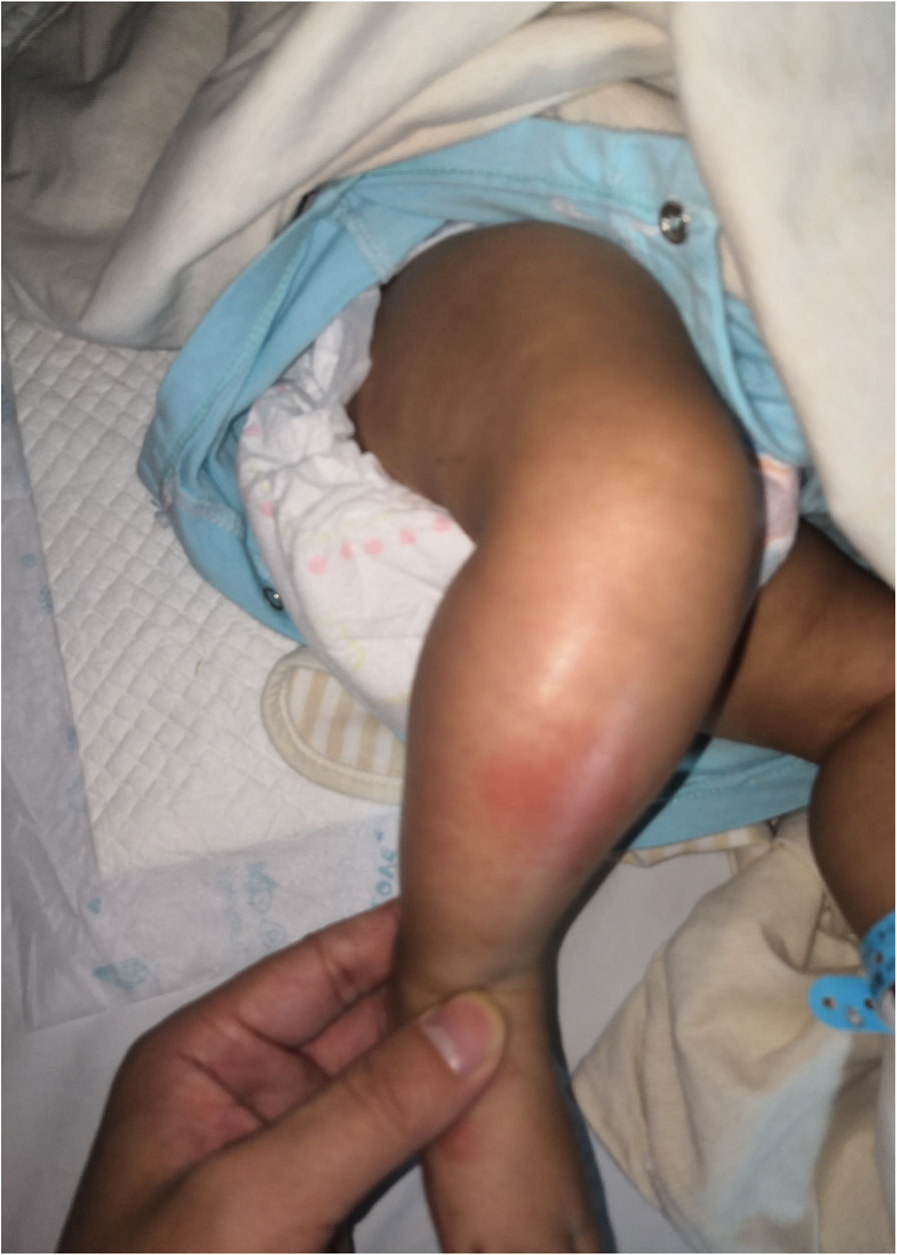
Swelling with erythematous changes in a 2 months old patient

A girl aged 2 months and 23 days had fever and swelling in her right lower leg and refused to move her right lower leg for 7 days. The patient’s fever subsided 5 days previously. Her body temperature ranged between 37.6 °C and 38.5 °C for 2 days. However, her right lower leg continuously swelled, with erythematous changes (Fig. 1). The patient would cry when someone pulled on her right lower leg. The patient was carried to our hospital by her parents. Through inquiry about the patient’s medical history, we found that the patient was delivered spontaneously and had no genetic diseases. The patient received a Bacillus Calmette-Guerin vaccination 10 days prior and was not being treated with antibiotics. The patient had no history of other diseases, such as sickle cell disease or leukemia. Physical examination revealed erythema, warmness, and tenderness on her right leg. When move her right knee, she grimaced.

The patient had a normal white blood cell count (WBC); erythrocyte sedimentation rate (ESR); and C-reactive protein (CRP), procalcitonin (PCT), and bone alkaline phosphatase (BAP) levels and a negative anti-tuberculous immunoglobulin G result (TB-IgG) (Table 1).

**Table 1:** Laboratory tests

WBC	7.41*10^9/L
ESR	13 mm/h
Hs-CRP	2.74 mg/L
HGB	110 g/L
PLT	590*10^9/L
ALP	442 U/L
BAP	126.30 μg/L
25-OH-VD	79.7 nmol/L
PCT	0.088 ng/ml
TB-IgG	Negative
Acid-fast staining	Negative
Blood culture	Negative

*WBC* White blood cells, *ESR* Erythrocyte sedimentation rate, *Hs-CRP* High sensitive C-reactive protein, *HGB* Hemoglobin, *PLT* Platelets, *ALP* Alkaline phosphatase, *BAP* Bone specific alkaline phosphatase, *25-OH-VD* 25 hydroxyl vitamin D, *PCT* Procalcitonin, *TB-IgG* Anti-Tuberculous-immunoglobulin G.

**Fig. 2:**
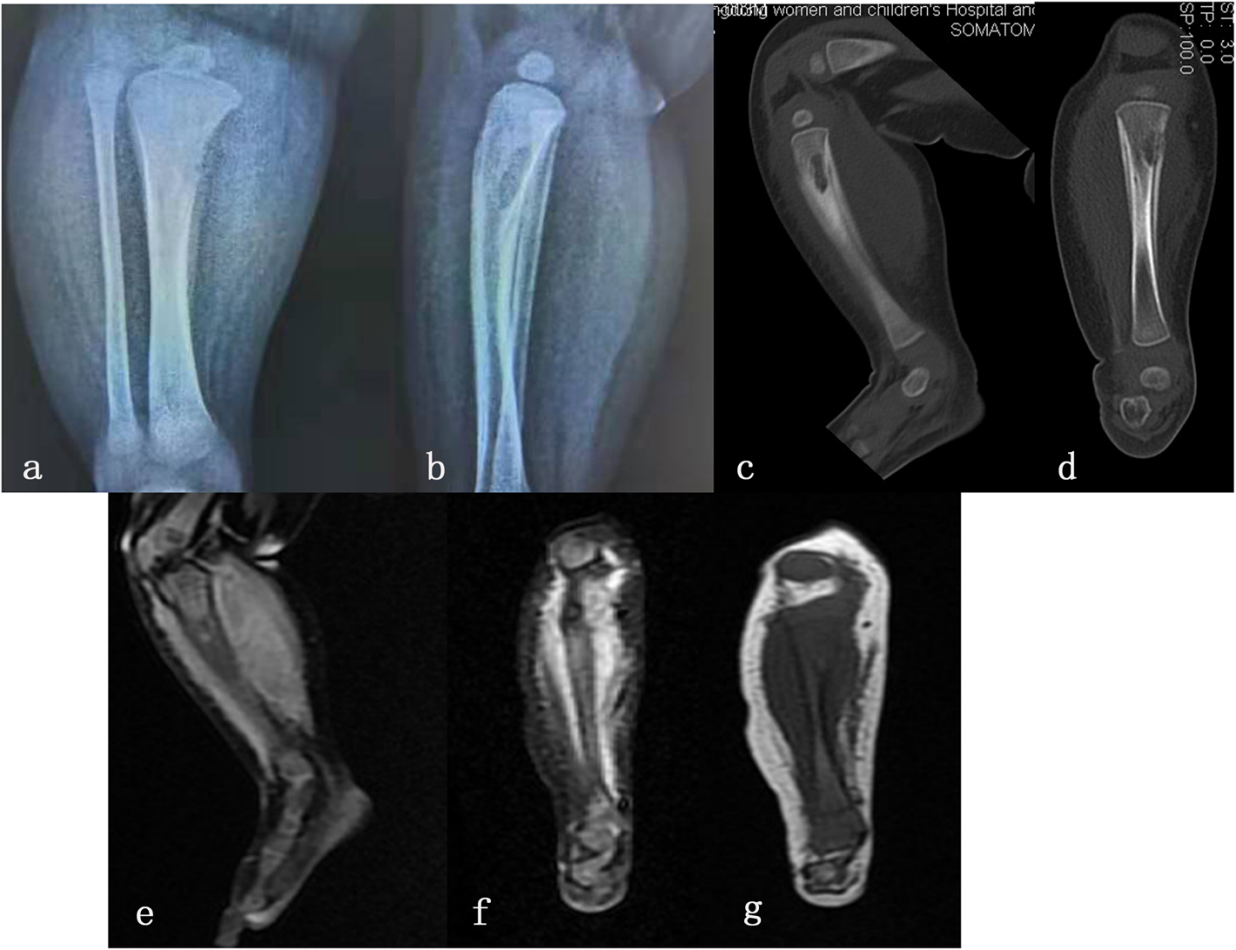
**a** and **b** Radiographs demonstrated a lytic lesion with periosteal reaction in proximal tibia in a 2 months old patient. **c** and **d** CT coronal and sagittal plane revealed a moth-eaten, ice melting sign, irregular erosions on her tibial metaphysis. **e**-**g** T1-weighted MRI confirms the processes of hypointense under the proximal tibial physis corresponding to hyperintensity on T2-weighted images with cortical breach and adjacent soft-tissue abscess

X-ray examination of her tibia revealed a poorly defined, irregular osteolysis and a moth-eaten pattern and showed a periosteal reaction in the proximal metaphysis of the tibia with adjacent new bone formation. Computerized tomography (CT) in the coronal and sagittal planes revealed moth-eaten and melting signs and irregular erosions on her tibial metaphysis. Cross-sectional CT revealed bone cortex lesions, indistinct borders, and laminated periosteal reactions over a large area. Magnetic resonance imaging (MRI) of the tibia showed a T1 hypointense region in the proximal tibia corresponding to an area of hyperintensity on T2-weighted images and a large subperiosteal abscess. MRI revealed periosteal edema, periosteal thickening and adjacent muscle edema (Fig. 2).

**Fig. 3:**
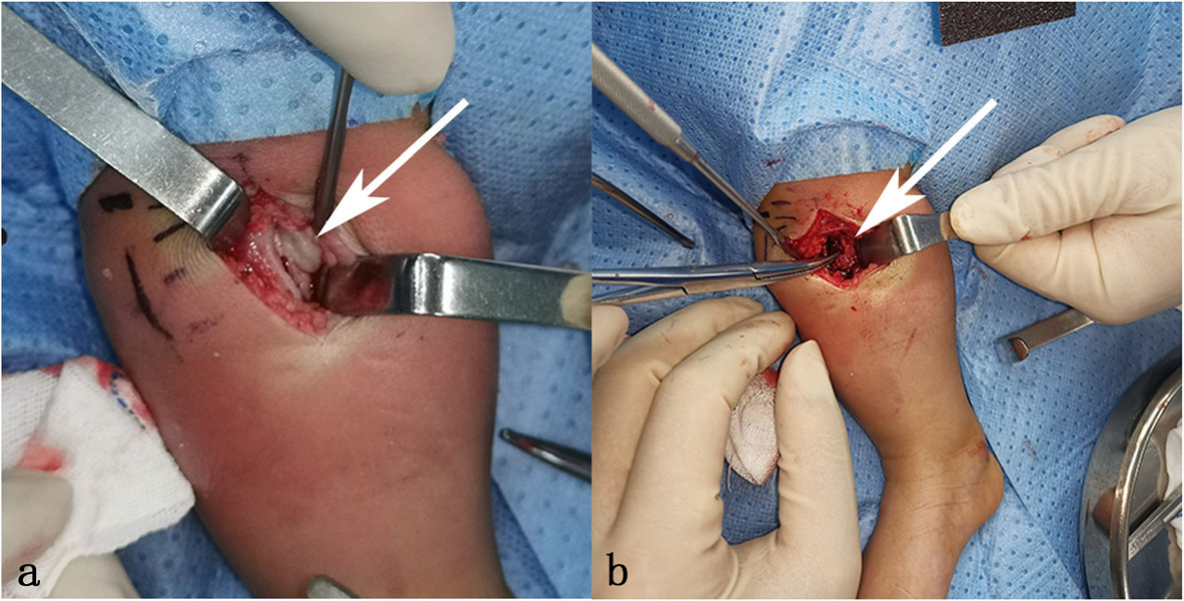
**a** Thicken periosteum in proximal tibia(white arrow) **b** Bright red tissue in medullary cavity(white arrow)

The medical history and imaging data led to a diagnosis of chronic osteomyelitis that had transformed from acute osteomyelitis due to delayed therapy. However, the serum inflammatory markers and the age of this girl did not support the diagnosis of chronic osteomyelitis. It was difficult to make an accurate diagnosis of osteomyelitis or bone tumor. Therefore, surgical biopsy was performed in this patient. A medial proximal tibial incision was made to reveal the lesions. Proximal tibial metaphysis periosteal thickening and edema were clearly visible intraoperatively. The tibial metaphyseal cortex was found to be defective upon incision of the periosteum. The metaphysis was adequately debrided by the creation of a 1 cm*1 cm cortical window to enable access to this region. Bright red tissue was seen in the subperiosteal space and within the metaphysis of the tibia (Fig. 3). We debrided the bright red tissue and sent it, as well as the periosteum, for biopsy and bacterial culture. After extensive debridement, we sutured the periosteum and skin and did not place surgical drains.

The patient did not receive intravenous antibiotics or oral antibiotics postoperatively. Fever and leg swelling and erythema resolved. The WBC and ESR as well as CRP and PCT levels were still normal post operation. Cultures of samples that were intraoperatively collected from the wound were negative. It took 2 weeks to confirm chronic inflammation on histological examinations performed by 3 institutions considered authorities in pathological identification.

The patient’s incision healed well and she was released from the hospital without incident. At the last follow-up visit at 12 months post operation, the patient had no pain or limitation in walking. She had no physical or radiographic sequelae.

## Discussion and conclusion

AHO occurs mostly in childhood and is caused by bacterial seeding that is thought to be due to transient bacteremia [10, 13]. In contrast to AHO, chronic hematogenous osteomyelitis (CHO) is caused by long-term infection that is thought to develop due to the persistence of microorganisms, low-grade inflammation, and the presence of dead bone (sequestrum) [14, 15]. Theoretically, it is possible for acute osteomyelitis to induce bone necrosis and progress to chronic osteomyelitis within 10 days [16]. In our case, a two-month-old girl progress to chronic osteomyelitis only in 7 days. Previous articles concentrated on the epidemiology, pathogenesis, diagnosis and therapy of acute hematogenous osteomyelitis [13, 17–19]. Several articles presented surgical and treatment methods for chronic osteomyelitis, such as debridement and the application of local antibiotics and treatments [11, 12, 20]. To date, few articles have reported the minimum age of chronic hematogenous osteomyelitis patients. This is the first case report chronic hematogenous osteomyelitis in a patient this young.

Diagnosing osteomyelitis was difficult because a causative organism was not identified. Zhorne DJ et al. reported that the positive culture rate was 86% in operative biopsy specimens and 43% in interventional radiology biopsy specimens without pretreatment with antibiotics in a retrospective evaluation of 67 infants and children (60 days to 18 years old) diagnosed with AHO [21]. Rebecca L Floyed et al. reported 40 cases of culture-negative osteomyelitis with initial presentations that were different than those in culture-positive cases and managed as presumed staphylococcal disease with excellent long-term results [4]. In our case, culture of intraoperative samples was negative even though there was no pretreatment with antibiotics. This may be explained by the following: 1. lower serum inflammatory markers have been associated with a higher possibility of negative culture result [22]; or 2. some bacterial species, such as *K. kingae*, do not grow on routinely used media, and we did not send the specimen for identification by polymerase chain reaction [23, 24]. Osteomyelitis is likely to be present when there are typical clinical and radiographic features of osteomyelitis along with a response to antibiotics in the absence of a positive culture. Peltola and Vahvanen [25] suggested that osteomyelitis be defined by the presence of two of the following criteria: pus aspirated from bone; positive bone or blood culture results; classic symptoms of localized pain, swelling, warmth, and limited range of motion in the adjacent joint; or radiographic changes typical of osteomyelitis. In our case, although undiscernible tissue aspirated from the bone was negative on bone culture, classic symptoms and radiographic changes helped us confirm the diagnosis of osteomyelitis.

**Fig. 4:**
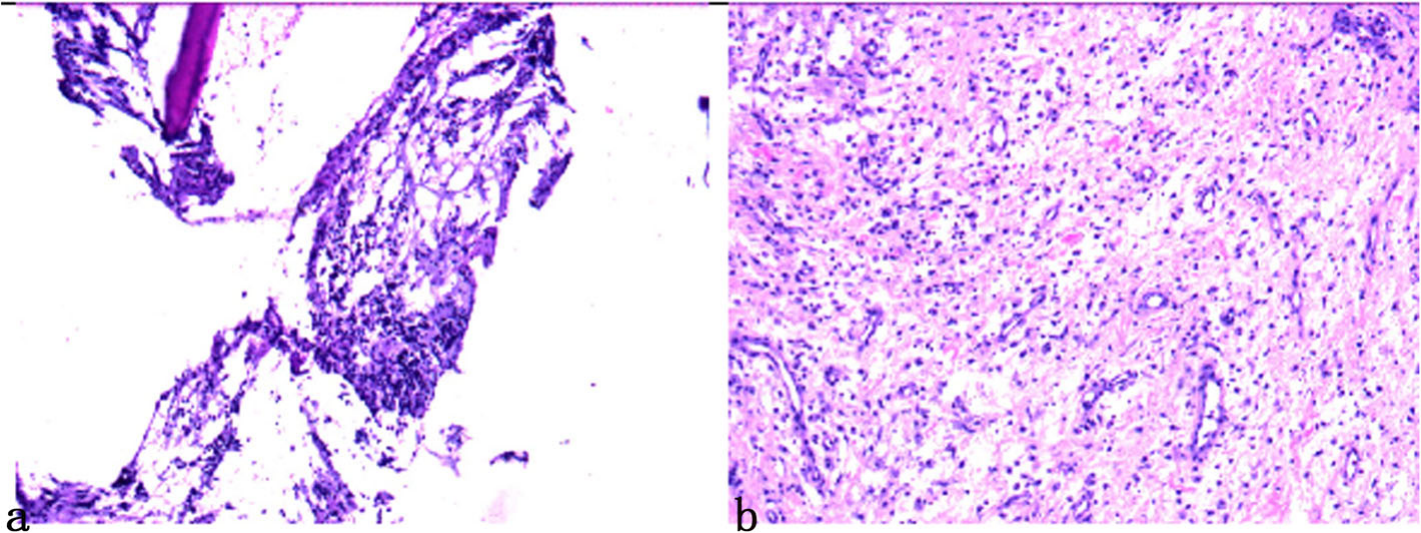
Pathological section of bone marrow and periosteum. **a** Bone marrow: fibrous hyperplasia and multinucleated giant cell reaction. **b** Periosteum: be infiltrated by lymphocytes, histiocytes, and plasma cells

The histological findings of chronic osteomyelitis are areas of woven bone and fibrosis with large numbers of lymphocytes, histiocytes, and plasma cells in the absence of neutrophils [26]. In our specimen, the bright red tissue was osteoblastic with hyperplasia, fibrous hyperplasia and multinucleated giant cells in bone trabeculae on histological examination. The periosteum had been infiltrated by lymphocytes, histiocytes, and plasma cells. The immunohistochemistry results were as follows: CD3(+), CD20(+), CD68(+), CD34(+), Desmin(−), and Ki-67(20%). Therefore, the histology results supported the diagnosis of chronic osteomyelitis (Fig. 4).

Surgical debridement is indicated for chronic osteomyelitis in association with bone abscess or destruction. A long-term course of parenteral antibiotics for 6 to 8 weeks is often appropriate. In our case, we did not treat this patient with antibiotics postoperation. The reasons were complicated. First, the bright red tissue obtained from the tibia intraoperative focus led the surgeon to arbitrarily diagnose bone tumor.

**Fig. 5:**
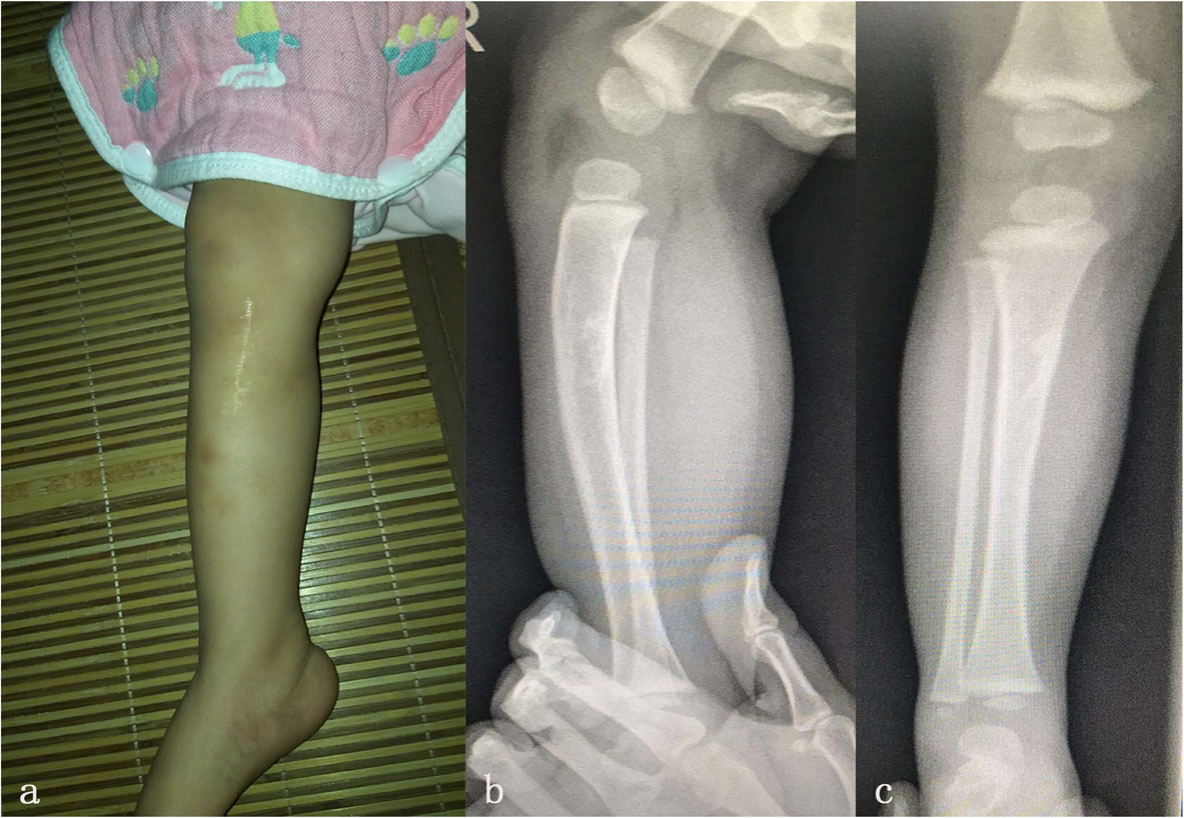
Appearance and radiographs 1 year postoperative

Second, the patient has no fever and has a normal WBC, ESR, CRP, PCT postoperation. In addition, when the pathology results come out, the patient has a good recovery. Finally, the patient’s incision healed well, and she was released without incident. At the last follow-up visit at 12 months post operation, the patient had no pain or limitation in walking. She had no physical or radiographic sequelae (Fig. 5).

In conclusion, we report a case of culture-negative chronic hematogenous osteomyelitis in the distal tibial metaphysis a 2-month-old girl. To the best of our knowledge, this is the first published case report of chronic osteomyelitis in a patient this young.

## Data Availability

All data generated or analysed during this study are included in this published article [and its supplementary information files].

## Abbreviations

AHO: Acute Hematogenous Osteomyelitis
CHO: Chronic Hematogenous Osteomyelitis
ESR: Erythrocyte Sedimentation Rate(ESR)
CRP: C-Reactive Protein
PCT: Procalcitonin
BAP: Bone Alkaline Phosphatase
TB-IgG: Anti-Tuberculous-immunoglobulin G
CT: Computerized tomography
MRI: Magnetic resonance imaging
ROM: Range of motion
